# Consideration of Mental Health and Well-Being in High-Level Sport: When Will a Coach-Centred Approach Be Introduced?

**DOI:** 10.1007/s40279-024-02044-x

**Published:** 2024-05-29

**Authors:** Chloé Leprince, Mathéo Maurin, Christopher Carling

**Affiliations:** 1 French Football Federation Research Centre, Clairefontaine-en-Yvelines, France; 2 Sport, Expertise and Performance Laboratory (EA 7370), INSEP, Paris, France; 3 Laboratory on Vulnerabilities and Innovation in Sport (L-VIS), University Claude Bernard Lyon 1, Villeurbanne, France

## Abstract

Coverage of problems relating to mental health and well-being is gaining ground in the sports sector today, both in the media and in the scientific literature. Despite exposure to numerous stressors and suffering from poor mental health, coaches have in general been largely overlooked in the scientific literature. Previous studies have mainly focused upon athlete populations. The absence of research means that there are real shortcomings in both understanding the mechanisms involved in the deterioration of coaches’ mental health and well-being and in the lack of specific support systems available. This paper first describes findings from the recent, albeit quite scarce, research investigating mental health and well-being in coaches. It then proposes a number of avenues for research and support protocols, both of which are currently ongoing at the French Football Federation Research Centre. The aim is to help support these key participants in the sports sector who arguably have not been given sufficient consideration until now.

## Key Points

**Table:** 

While mental health and well-being are receiving significant coverage in the scientific literature in the context of high-level sport, coaches are frequently overlooked in this area.
Given their extensive responsibilities and exposure, coaches are at significant risk regarding their mental health and well-being.
Our research group at the French Football Federation Research Centre has adopted an innovative scientific approach that aims to better understand the mental health and well-being of its football coaches and implement dedicated support programmes.

## Introduction

Concerns relating to mental health and well-being in sport have received wide attention in recent years although scientific research has arguably focused upon athletes [1, 2]. Indeed, two recent reviews have highlighted a limited amount of research investigations on these key issues in coaching populations [3, 4]. This observation implies that coaches are often omitted from the psychological monitoring and support programmes available in high-performance systems. Yet given their level of responsibility and exposure levels, this population is particularly at risk of a range of problems relating to their mental health and well-being. Apart from the recent, albeit quite scarce, research that highlights the difficulties encountered by coaches and their psychopathological effects [5–8], a comprehensive understanding of the mechanisms that cause, go together with, or are a consequence of the deterioration in their mental health and well-being is lacking [9]. Perhaps more importantly, few practical recommendations to help support coaches have been made [4, 10]. Indeed, support programmes for athletes are now much more developed and widely accepted than in coaching circles. In the face of the deterioration in coaching conditions and particularly in the context of association football (56.9% of football coaches were notably dismissed worldwide last season according to a 2023 CIES study [11]), it is arguably time to provide better support for this population regarding their mental health and well-being. Through developing research programmes related to this specific issue, our research group at the French Football Federation (FFF) Research Centre has made football coaches the focus of its work and support with the aim of helping them to perform optimally while benefiting from appropriate support programmes.

This paper first describes the results of the recent, albeit rare, research focusing upon the mental health and well-being of coaches. It then suggests a number of research avenues for reflection whilst also discussing the support protocols, together with the different tools, that the FFF Research Centre is currently rolling out for its coaches.

## Stressors and the Mental Health of Coaches: A Startling Observation

### Chronic Exposure to Stressors

Coaches particularly at elite standards encounter multiple stressors in their daily lives, which have already received attention in the scientific literature [12–14]. In a previous review, Norris et al. [14] identified various categories of stressors for coaches involved in a wide variety of sports. These are related to their own performance, that of their athletes, and other organisational, contextual, interpersonal, and intrapersonal factors (see Table 1 for more details). These stressors can resemble those experienced by athletes, such as the need to perform, or their expectations of others [14, 15], but also differ significantly, with stressors specifically related to athlete and team management (e.g. commitment, conflicts, player selection), job insecurity, and relations with organisational stakeholders. Among the numerous stressors identified, organisational ones (e.g. work overload, difficulties in striking a work–life balance, quality of the work environment) were identified as having a large impact on coaches, since these are cumulative and chronic, and lead to some long-term negative consequences [7]. Organisational stressors are also the most difficult to manage as coaches have less control over these when compared to others, such as personal ones [6]. Faced with exposure to multiple stressors, coaches may try to adapt by adopting coping strategies. Unfortunately, a recent study among football coaches showed that the numerous demands they face are generally perceived as negative and threatening, and many of the strategies subsequently applied to deal with stressful experiences are deemed ineffective [6]. The repeated confrontation with stressors deemed to exceed a coach’s self-resources can subsequently have negative consequences affecting, for example, their ability to achieve their objectives, the quality of their daily life, and their well-being [6].

**Table 1:** Presentation of the different types of stressors encountered by coaches with examples

Stressor category	Examples
Stressors related to athlete performance and injuries	Athlete’s coachability Athlete’s professionalism Athlete’s attitude Athlete’s commitment Athlete’s injuries
Stressors related to coach performance (i.e. their own performance)	Placing unrealistically high standards on themselves Their communication Quality of their decisions Self-criticism
Organisational stressors	Administrative tasks Finances Overload Environment Work–life balance Pressure and expectations for performance and results
Stressors related to coaches’ contextual experiences	Schedule Lack of resources Job security Age and experience Level of competition
Stressors related to interpersonal experiences	Social support Expectations of others (parents, medias, staff) Relationships with athletes
Stressors related to intrapersonal experiences	Performance outcome Lack of control

Inspired by Norris et al. [14]

### Mental Health and Well-Being of Coaches Compromised by Stressors

Among the few studies that have examined coaches’ well-being, Bentzen et al. [16] showed that a coach’s level of well-being decreases during a sporting season while exhaustion increases. Declining levels of well-being and greater exhaustion may be linked to a perceived lack of control in their professional activity [16]. This perception can be explained by numerous stressors that are, in part, uncontrollable (e.g. coaches cannot, by definition, train or perform in place of their athletes) [17, 18]. Job insecurity during the season also seems to contribute significantly to this deterioration since it is associated with a decrease in well-being and an increase in ill-being [19]. This insecurity, which is particularly high among coaches, is linked not only to expectations around results, but also to the low, precarious, and often short-term employment possibilities in this profession, as it is not always easy to find a comparable role at a similar standard of play following dismissal [19]. Confronting these stressors and their effects are fraught with consequences. For example, a coach who mismanages, feels overwhelmed by, or is unable to cope with difficult stress-related well-being problems can negatively impact their own performance. This in turn can negatively impact the coach–athlete relationship and the mental state of athletes, and subsequently harm their competitive performances [20].

Beyond their impact on well-being, coaches who have difficulty coping with the numerous stressors that they encounter can experience a deterioration in their mental health [5, 7]. Stressors and, specifically, dissatisfaction with social support and life balance are robust correlates of psychological distress [5]. While the literature has focused heavily on the mental health of athletes, it should be noted that coaches have similar prevalence rates for mental health disorders [21]. Research has shown that 55% of coaches from all sports and at all levels report that they have previously suffered from a mental illness [22]. The consequences can be worrying for coaches, as research reports high-risk alcohol consumption [5, 7], moderate to severe sleep disturbance [5], and anxiety and depressive disorders, which are the most common issues [7, 22, 23]. Given the close link between daily stress, which can be chronic and potentially unmanageable, and the probability of developing depressive symptoms [24], it appears necessary to account for and subsequently make attempts to attenuate the effects of these stressors through interventional measures to enhance coach support programmes.

### Rare and Mostly Non-specific Interventional Studies

In view of the difficulties encountered by coaches and their related consequences, several research proposals have been forwarded to help promote their mental health and well-being. These include support focused on the quality of professional relationships [22], work–life balance [22, 25], avoidance of ambiguity in roles [22], resilience [7], the ability to self-manage one’s workload [26], and autonomy in tasks [26, 27]. Regrettably, few interventional studies have been conducted in coach populations. Indeed, in their recent review, Breslin et al. [3] identified only six papers covering interventions in coaches (Table 2). In addition, the main aim identified by the authors across these six studies was, indirectly, to improve the well-being of athletes rather than that of the coaches themselves. It has been suggested that the early implementation of intervention programmes is necessary to detect and treat the onset of symptoms of mental health disorders in coaches, help them minimise stressors, facilitate positive assessments and emotions, and, ultimately, assist promotion of their well-being [5, 8, 28].

**Table 2:** Descriptive information on interventional studies involving coaches. Adapted from Breslin et al. [3]

Authors (year of study)	Study design; duration	Sample characteristics	Mental health descriptor; mode of delivery
Bapat et al. (2009) [31]	Pre-post design; 3 weeks	Sport club leaders (*n* = 40; age = 38.62; 16 males, 24 females)	Mental health literacy through mental health first-aid training; 8-h training programme delivered over three sessions using a range of presentations, tasks, and homework
Breslin et al. (2017) [29]	Controlled trial; 1 day (3-h session)	Sport coaches (*n* = 244; 126 males, 118 females)	Mental health awareness programme involving videos and discussions with athletes who have experienced depression; 3-h programme delivered in one session by a public health agency provider
Longshore and Sachs (2015) [33]	Controlled trial; 6 weeks	College coaches (*n* = 20; age = 34.5; 8 males, 12 females)	Mindfulness training programme to develop emotional awareness and reduce stress; an initial 1.5-h group session followed by a 6-week home programme
Pierce et al. (2010) [32]	Pre–post design (club leaders); controlled trial (football players); 3 weeks	Club leaders (*n* = 36; age = 45); and football players (*n* = 275; age = 21)	Mental health literacy; 12-h psycho-educational group sessions for leaders; information sessions were conducted with players alongside informal information
Sebbens et al. (2016) [30]	Controlled trial; 1 day (4 h)	Coaches, trainers, support staff and service providers (*n* = 166; age = 37.8; 83 males, 83 females)	Mental health knowledge and confidence programme; 4-h applied workshop involving case studies, role-playing and videos
Vella et al. (2020) [61]	Controlled trial; 8 weeks	Adolescent male sport participants, parents of participants and coaches (*n* = 1004)	Mental health literacy: multi-component sports-based programme to promote early intervention, help-seeking and resilience

In general, interventional studies concerning coaches’ mental health have been shown to increase their knowledge of mental health disorders, make them more confident in helping others with a mental disorder, and bring about change in attitudes that stigmatise those with mental health disorders [29–32]. However, none of this research appears to have specifically targeted the coaches’ own well-being, and to what extent they could improve their ability to care for their own mental health. Longshore and Sachs [33] for example, demonstrated that a 6-week mindfulness programme can have positive effects in coaches. Improved emotional control, lower levels of anxiety and unproductive emotional states, and greater curiosity were notably observed. This sport-specific intervention can help lead to positive adjustments, balance, and psychological well-being in all areas of a coach’s life, through better work–life balance among other factors [33]. A programme of conscious self-reflection has also shown positive effects, with greater commitment in coaches towards their well-being and improved self-awareness and self-compassion [34]. Despite some encouraging research, there are very few studies in proportion to the magnitude of the issues at stake (described above) regarding the well-being and mental health of coaches. As such, additional research is required to test and roll out instruments and protocols adapted to coaches’ lifestyles, and ultimately help them deal with these largely unresolved issues.

## Ongoing Research at the FFF to Improve Overall Understanding of the Mechanisms Involved in the Deterioration of Mental Health and Well-Being of Coaches

The recent aforementioned studies have mainly highlighted the stressors that coaches encounter in their professional activities and the possible consequences on their mental health and/or well-being. These studies have nevertheless produced essential findings that have shed light on these issues, which have frequently been overlooked until recently. However, there are limitations, which will be discussed below (Sects. 3.1 and 3.2), that do not enable a comprehensive understanding of the mechanisms underlying the deterioration of coaches’ mental health, and additional research is warranted. The FFF Research Centre study group has developed a structured programme to explore several lines of research based upon (1) recommendations to conduct longitudinal studies to track changes in mental health and well-being throughout a season [9], and (2) collection and analysis of a range of variables to enable the most comprehensive understanding possible of the mechanisms underpinning deterioration in the mental health and well-being of coaches.

### Longitudinal Experimental Designs to Monitor Coaches Throughout the Season

Among previous studies conducted on the mental health and well-being of coaches, few have utilised longitudinal designs [35] to monitor changes in levels of mental health and well-being over time [36], which is a major limitation [7, 8]. Furthermore, the variables and disorders associated with the mental health and well-being of coaches, such as sleep [5], at-risk alcohol consumption [5, 7], eating disorders, and psychoactive substance disorders [23], are typically measured in isolation and transversally, thereby limiting a holistic understanding of the influence and cause–effect relationships of the various interacting factors. The level of social support, an important stressor [14] and a robust correlate of the psychological distress of coaches [5], has also been investigated [37, 38]. Unfortunately, up to now, this support has again been studied in isolation to the other factors mentioned above. To overcome these limitations and improve understanding of the challenges of the mental health and well-being of coaches, the FFF Research Centre is currently conducting several studies in professional and amateur football coaches. These investigations are employing longitudinal methodologies, making it possible to monitor coaches systematically over the entire course of a competitive season. The tracking begins before the summer break, when the coach returns from vacation, then every month until the end of the season. This longitudinal experimental design also utilises a pragmatic mixed methods combination [39]. Quantitative data derived from questionnaires (described in Sect. 3.2) and analyses of context across the competition season (notably the results of their team) in combination with qualitative data from interviews are used to investigate and identify in greater depth the coaches’ “key” moments across the season and their personal experiences.

### Holistic Consideration of the Various Influencing Factors to Ensure a Greater Understanding of Mental Health and Well-Being of Coaches

The need to explore the environmental, personal, and relational factors that influence the well-being of coaches has been highlighted [40]. As such, the studies conducted by the FFF Research Centre are currently investigating a range of variables to help decipher the mechanisms via which these different factors can affect mental health and well-being over the course of a season. Variables such as vitality, defined as “positive feeling of having energy available to the self” [41]), sleep, diet, social support, and personal practices (the time coaches devote to rest and recover) are measured concomitantly alongside mental health and well-being to help understand the roles of various factors when a deterioration is observed. Finally, dispositional measurements are also being used to identify profiles that may potentially be more at risk than others (e.g. personality traits [42], type of passion [43], emotional competences [44]). Through the various methodologies used and variables investigated, our research group aims to pinpoint the dynamics by which coaches observe a decline in their mental health and well-being over the course of a season, as well as the protective or degrading role of certain factors.

## Suggestions for Practical Applications: An Example of Support Offered to Coaches

Pending the scientific results from its aforementioned longitudinal monitoring that aims to enrich coach support programmes, the FFF’s research group runs both individual and collective sessions with coaches to raise their awareness of the importance of mental health and well-being regarding the effectiveness of their own performance. These sessions are notably built into the federation’s coach education pathways to avoid any possible stigma that such topics may potentially raise when coaches need to seek help [28]. Psychologists and researchers from our research group consequently intervene during coaching education courses to address the question of mental health and well-being in this population. In addition, as coaches have prominent leadership roles and play a key role in cultivating their organizational or team environment, any positive effects on them may in turn influence the system in which they evolve, notably by shaping cultural and institutional attitudes towards mental health [10].

### An Approach Designed to Optimise Personal Resources

Working on mental health and well-being via research and coach education programmes requires a posture and objectives that are both clear and consistent within these contexts. The objectives of the FFF Research Centre are not the analysis of symptoms or therapeutic care but rather the optimisation of personal resources to help develop growth processes [45, 46]. This objective coincides with the precepts of positive psychology, defined as the scientific study of factors promoting the well-being and development of individuals and groups [47]. In this theory, mental health is described based on the World Health Organization (WHO) definition [48], which addresses this concept as a state of well-being in which a person can realise their abilities, cope with the normal stresses of life, perform productive work, and contribute to the life of their community. Well-being is studied in its subjective (hedonic well-being associated with pleasure) and psychological (eudemonic well-being associated with meaning) dimensions [49].

Thus, the aim of our support programmes is to help coaches identify the factors underpinning the improvement of their mental health and well-being and to improve awareness of the resources at their disposal to cope with the demands inherent to their personal and professional environments. Furthermore, the concept of “restoration”, as described by Hartig [50] in the context of restorative environments, is central to this support for coaches. These restorative environments are described as having therapeutic potential, enabling the renewal of the cognitive, emotional, physical, and social resources that are naturally depleted during an individual’s adaptive efforts [50]. Our work is thus positioned *downstream* of the adaptive efforts inherent in the professional and personal life of coaches, by aiding them to renew their resources to help them to cope, in the long term, with the pressures associated with their profession (e.g. media pressure, performance challenges, organisational stressors), which they themselves cannot always influence directly. Consequently, our support for coaches encompasses three areas: (1) exposure to restorative environments, (2) restorative experiences, and (3) restorative interactions. These are discussed in turn.

### Exposure to Restorative Environments

Humans have always been aware of the impact of their environment on the way they live and act. In recent years, however, several scientific studies have increased understanding of the role of the environment. As a result, the concept of the restorative environment [50] has emerged in the literature. This is described as environments with therapeutic potential that contribute to increased mental health, well-being, and performance. In sport contexts, several studies have highlighted the importance and benefits of natural environments for the mental health and well-being of athletes [51, 52] and inferences can be made for coaching populations. Indeed, the FFF Research Centre has set up its own restorative environment. Surrounded by nature (an environment with very high restorative powers – see Ohly et al. [53] for a meta-analysis), the research centre facility was also carefully designed regarding the choice of colours, materials, sounds, shapes, and smells (i.e. positive distractors [54]). The aim is to ensure coaches benefit from a “restorative environment experience” to make them aware of the impact that the places in which they live, work, hold meetings, participate in seminars, and so on has on them and on their athletes. This experience can then eventually be employed as a model to inspire coaches to develop the restorative power of their own work environments.

### Restorative Experiences

Exposure to restorative environments can only have a limited effect if coaching practitioners do not benefit from restorative experiences within those environments. We consider as “restorative” any experience set up by the coach to replenish their resources, give them pleasure, nourish their enthusiasm, and reconnect them to their physical and emotional sensations. In this respect, our research centre offers several activities including mind–body practices such as Garuda [55], breathing techniques [56], meditation techniques [57], music-based digital therapies [58], and even movement through sports and fun activities such as trampolining [59]. The research centre’s aim and role is to determine which of these instruments and techniques are best suited to a coach’s personal needs, and how to support them with their day-to-day application (when, in which combination, at what frequency, for which purpose, and how to deal with and adapt these to the time constraints specific to their profession, etc.).

### Restorative Interactions

The final factor conducive to renewing a coach’s resources is the quality of their interpersonal relationships that develop within the restorative environments. Given the importance of relationship factors in coaching and the role of social support frequently cited in studies dealing with coaches’ mental health and well-being [5, 7, 14, 22], it seems important to specifically account for interpersonal aspects, both professionally (optimising communication and the general atmosphere within a team or a group of players) and personally (maintaining a good life balance and high-quality relationships with friends and family).

Didymus et al. [60] highlighted the importance of empowering coaches to ensure that they consider their own mental health and well-being. As such, the support programmes proposed by the FFF Research Centre aim to help coaches become more self-reliant by increasing their self-awareness through identification of their needs and how they function. By raising their awareness and providing them with a “toolkit” to help them learn how to take care of themselves (through their environment, experiences, and restorative interactions), the research centre’s support policies encourage coaches to draw upon their personal and external resources. The aim is to help coaches to “replenish themselves” daily, and to help ready them to cope with the short-, medium-, and long-term challenges that will shape their coaching career.

## Conclusion

Mental health and well-being have received an ever-increasing amount of attention in sport. However, up to now, studies have largely been confined to athlete populations and coaches have frequently been overlooked. Given the complexity of their occupation and the findings that have nevertheless recently emerged regarding its impact on their mental health and well-being, additional research is necessary to help understand and elucidate the reasons and mechanisms underpinning any deterioration. The results of these explanatory studies could then be used to deploy more targeted, and potentially more effective, interventional studies to help promote the mental health and well-being of coaches over the short, medium, and long term. The FFF Research Centre has opted for this scientific approach and is currently conducting studies in coaches that aim to increase understanding of, and subsequently help confront, these problems and ultimately optimise support programmes to aid personal and professional performance.
